# Bilateral strength balance of knee extensor and flexor muscles in female soccer players

**DOI:** 10.3389/fphys.2025.1681804

**Published:** 2025-10-03

**Authors:** Oscar Andrades-Ramírez, David Ulloa-Díaz, Luis Romero-Vera, Bryan Alfaro-Castillo, Gustavo Muñoz-Bustos, Carlos Jorquera-Aguilera, Claudio Carvajal-Parodi, Luis-Javier Chirosa-Ríos

**Affiliations:** ^1^ Department of Physical Education and Sport, Faculty of Sport Sciences, University of Granada, Granada, Spain; ^2^ Facultad de Educación y Ciencias Sociales, Universidad Andres Bello, Entrenador deportivo, Concepción, Chile; ^3^ Department of Sports Sciences and Physical Conditioning, Universidad Católica de la Santísima Concepción, Concepción, Chile; ^4^ Facultad de salud y ciencias sociales, Universidad de Las Américas, Licenciatura en ciencias de la actividad física, Concepción, Chile Facultad de Humanidades y Educación, Concepción, Chile; ^5^ Physical Education Department, Facultad de Humanidades y Educación, Universidad de Atacama, Copiapó, Chile; ^6^ Facultad de Salud, Escuela de Nutrición y Dietética, Universidad Santo Tomás, Iquique, Chile; ^7^ Facultad de Ciencias, Escuela de Nutrición y Dietética, Universidad Mayor, Santiago, Chile; ^8^ Facultad de Ciencias de la rehabilitación y Calidad de vida, Escuela de kinesiología, Universidad San Sebastián, Concepción, Chile

**Keywords:** muscle strength, knee flexors, knee extensors, dynamometer, female soccer, lower limbs

## Abstract

**Background:**

The aim of our study was analyze the bilateral strength balance of the knee extensor and flexor muscles in female soccer players.

**Methodology:**

Participated in this study twenty-three professional female soccer players. The volunteer participants of the study were eligibly if: (a) signing of informed consent, (b) 5 years of sporting experience as a soccer player and have experience with training and evaluation of muscle strength in the lower limb, (c) participate in five weekly training sessions (d) no musculoskeletal pathology in the lower limbs 6 months prior to the evaluation date. The assessment was performed unilaterally, with peak muscle strength values recorded using the FEMD device software at a constant velocity of 0.4 m s^-1^. The range of motion (ROM) was 90°–0° of joint extension in the sitting position and 150°–90° of flexion in the prone position. Each participant was required to perform their maximum effort for all repetitions.

**Results:**

Bilateral strength balance measurements were obtained in the range of 10.68%–13.80% for maximum muscle strength in knee extension and 13.27%–15.21%. No significant differences (*p* > 0.05) were found in the comparison of independent means for maximum muscle strength between the knee extension and flexion in the concentric and eccentric phases. Significant differences (*p* < 0.01) and small ES (ES < 3.32) were found in peak muscle strength measurements of the dominant and non-dominant lower extremity in the unilateral comparison of the extensor muscle and the flexor muscle group in the concentric and eccentric phase.

**Conclusion:**

In the analysis of bilateral strength balance, lower indices are presented in the knee extensor compared to the flexor, these bilateral indices would reveal intrinsic and extrinsic risk factors for musculoskeletal injuries in the hamstring muscles and anterior cruciate ligament of professional soccer players.

## 1 Introduction

The soccer players perform activities involving rapid movements such as jumping, sprinting, contact with opponents, changes of direction (COD) and technical actions (e.g., shooting, passing, etc.), which require fast and powerful movements involving the lower limb muscle groups to perform actions characterized by maximum intensities and rapid speed that are important during training and competition periods (AlTaweel et al., 2022; Beato et al., 2021; Pecci et al., 2023). For sports actions to have a better performance, an assessment and analysis of muscle strength is necessary, which has been of great interest in the fields of physical rehabilitation, sports performance and prevention of sports injuries (Donskov et al., 2021; Janicijevic et al., 2023; Ruas et al., 2019; Venegas-Carro et al., 2022). Among the muscle groups that make up the lower extremities, the muscles that make up the quadriceps and hamstrings have a fundamental anatomical and biomechanical role in the knee and hip, due to the production of muscular force related to the movements of soccer (Morin et al., 2015).

The assessment of muscle strength in soccer players, especially that of the quadriceps and hamstrings, is essential because it provides biomechanical and performance information on the lower limbs (Keytsman et al., 2024). One of the muscle strength assessments used for the lower limbs is bilateral strength balance (BSB), due to the relationship between the maximum strength of the dominant leg, which can be defined as the preferred limb for performing specific motor movements in sports (Clemente et al., 2022) or for kicking a ball (Maly et al., 2016), and the non-dominant leg, which has less coordination skills than the dominant leg (Fort-Vanmeerhaeghe and Romero-Rodriguez, 2013). These asymmetries between the lower extremities in male and female soccer players should be less than the 10% limit suggested (Bishop, Coratella, et al., 2021; Loturco et al., 2016) and another study suggests that they should be less than 15% (Zimermann et al., 2020). This analysis provides information on muscle weakness and imbalance, which are injury risk factors that must be considered in sports performance (Helme et al., 2021; Saryono et al., 2022). Because the skills developed in soccer are unilateral and require asymmetric movements and patterns, which increases asymmetric musculoskeletal adaptations in the lower limbs (Rutkowska-Kucharska, 2020).

For sport science research inter-extremity asymmetry is not a new concept, a systematic review Bishop et al. (2018), indicates a high prevalence of asymmetry between limbs, these asymmetries in muscle strength negatively affect physical performance and sport specific skills, decreasing these differences between limbs seems to be favorable for female soccer players. Previous studies (Pardos-Mainer et al., 2020), and Pardos-Mainer et al. (2019), reported the existence of imbalances and asymmetries in lower extremity muscle strength in female soccer. In the studies (Menzel et al., 2013; Lockie et al., 2014), found that functional asymmetries not only lead to an increased likelihood of injury, but also to decreased performance. In another the study (Skalski et al., 2024), it was shown that female soccer players with less muscular imbalance exhibit better performance in straight-line sprints of 20 m and 30 m.

In a previous study, Contreras-Díaz et al. (2023), evaluated the muscle strength profile of the hip muscles in soccer players according to their position on the playing field with functional electromechanical dynamometry (FEMD), Multi-joint device that allows for the assessment of different forms of strength in a linear isokinetic manner, incorporating a cable wound on a reel located within the device. Modalities without displacement (isometric and vibratory) and with displacement (tonic, kinetic, elastic, inertial and conic) can be used through a stable or variable speed/resistance (Jerez-Mayorga et al., 2020), reporting values in BSB that are above 15%. Therefore, a study analyzing BSB and the muscle strength profile of quadriceps and hamstrings in professional female soccer players controlled with FEMD is needed.

In relation to the background presented, the primary objective of our study was to analyze the bilateral strength balance of the knee extensor and flexor muscles in female soccer players, secondly, to present an analysis of bilateral and unilateral maximum muscle strength of the knee extensor and flexor muscles. As a hypothesis, it is proposed that there is a bilateral strength imbalance of the knee extensor and flexor muscles in female soccer players, in addition, to a difference in bilateral and unilateral maximum strength.

## 2 Materials and methods

### 2.1 Study design

A cross-sectional study design was used with non-probabilistic sampling by convenience. Participants in this study were scheduled to attend two familiarization sessions. In the first session, we assessed anthropometric measurements and familiarized participants with the use of the FEMD device. In the second session, the motor gesture of the muscular strength evaluation was performed with the FEMD with a load of 10% of the body weight. For evaluations of peak muscular strength Concentric (CON) and eccentric (ECC), we use the dependencies of the controlled motion laboratory of the Universidad Católica de la Santísima Concepción, Concepción, Chile.

### 2.2 Participants

Participated in this study twenty-three national-level professional female soccer players at the start of the season (The demographic information of the participants is presented in Table 1), before starting the study, the players and the coaching staff were informed of the benefits and possible risks of performing the evaluation. There was no blinding or randomization of participants. It is recommended not to ingest analgesic substances or substances that alter metabolism before or during the evaluation. The volunteer participants of the study were eligibly if: (a) signing of informed consent, (b) 5 years of sporting experience as a soccer player and have experience with training and evaluation of muscle strength in the lower limb (in order to control the learning bias and so that the athlete knows the maximum muscular strength work), (c) participate in five weekly training sessions (d) no musculoskeletal pathology in the lower limbs 6 months prior to the evaluation date. Participants who did not complete the assessment protocol were excluded from the study (n = 1). The goalkeepers were excluded due to differences in physical training with the field players. The study protocol and methodology were reviewed and approved the Ethics and Research Committee of the Universidad Católica de la Santísima Concepción N° 01/2024 (approved 01 April 2024) and was conducted following the Declaration of Helsinki (World Medical Association, 2013).

**TABLE 1:** Demographic data of the participants.

Demographic characteristics	Mean		SD	
Age	19.08	±	1.16	years
Weight	58.04	±	6.39	height
Height	1.61	±	0.04	meters
BMI	22.24	±	2.01	kg/m^2^

SD, standard deviation; BMI, body mass index; kg, kilograms; m, meters.

### 2.3 Materials

Participants in this study had their muscle strength assessed using the FEMD measuring device (Dynasystem, Model Research, Granada, Spain). The device has a system accuracy of 3 mm for displacement, 100 g for the sensitivity of the detected load, a velocity range between 0.05 m s^−1^ and 2.80 m s^−1^ and a sampling frequency of 1000 Hz. The precision of the force and linear velocity is controlled and regulated by a 2000 W electric motor. In addition, it has a load cell that detects the tension applied to the rope and the signal is transmitted to an analog-digital converter with a 12-bit resolution. Speed and displacement data are collected with a 2500 ppr encoder attached to the roller inside the device. The various sensors obtain information at a frequency of 1 kHz. A previous study evaluated the validity of the FEMD with a linear velocity transducer (T - Force), obtaining almost perfect correlations (r < 0.99) for velocities (0.40, 0.60, 0.80, 1.00, and 1.20 m s^−1^ (Rodriguez-Perea et al., 2021) and intra-sets reliability measures were obtained, reporting acceptable absolute reliability (CV% < 9.71) and extremely high relative reliability (ICC = 0.92–0.98) for the motor gesture of knee extension and flexion for velocity 0.40 m s^−1^ (Andrades-Ramírez et al., 2025).

### 2.4 Assessment of peak muscle strength

It is recommended not to ingest analgesic substances or substances that alter metabolism before or during the evaluation. The muscle strength assessment session begins with a general warm-up that was performed with (a) 5 min on a stationary bicycle at 60% of the heart rate reserve, hip, knee and ankle joint mobility (5 min), lower limb strength exercises with body weight bridges hamstrings and squats (5 min); (b) a specific 15-min warm-up with knee extension (seated position) and flexion exercises (prone position) that would subsequently evaluate muscular strength at a submaximal load at the selected speed with a load of 10% of body weight, It was requested that they be carried out two sets of three repetitions with 3 minutes of rest between each of the sets were used as volume. Subsequently, each participant was asked to perform their maximum effort in all the repetitions they had to perform. The assessment was performed unilaterally, with peak muscle strength values recorded using the FEMD device software at a constant velocity of 0.4 m s^−1^ (Rodriguez-Perea et al., 2021), in the CON phase movement in which the selected range of motion (ROM) must be reached until the end and an ECC phase that attempts to oppose the movement throughout the selected range backwards which is considered a repetition.

**FIGURE 1:**
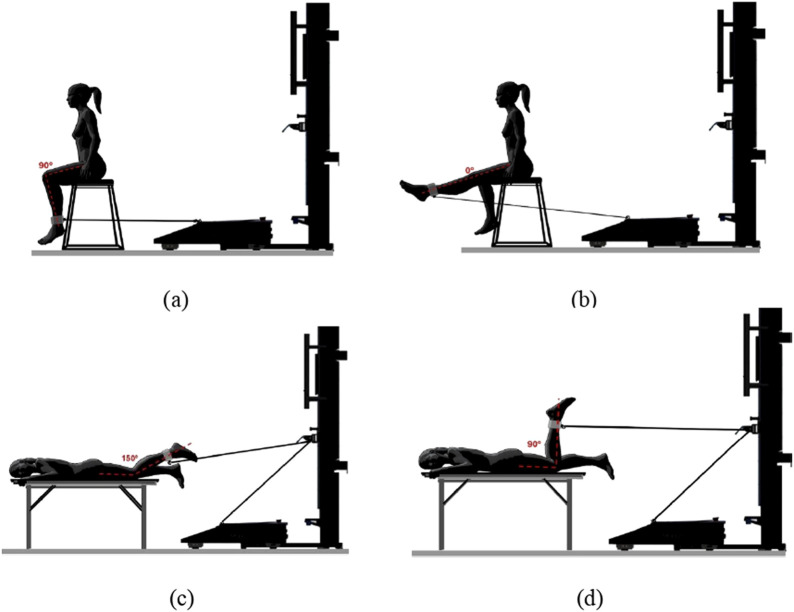
Assessment of peak muscle strength of the knee extensor muscles: **(a)** initial position; **(b)** final position. Assessment of peak muscle strength of the knee flexor muscles: **(c)** initial position; **(d)** final position.

Knee joint position was measured by a physiotherapist using a hand-held clinical goniometer. A ROM for a range of joint extension of 90°–0° in the sitting position (angulation between the center of the hip joint, knee joint, and lateral ankle malleolus) and flexion of 150°–90° in the prone position (angulation between the center of the hip joint, knee joint, and lateral ankle malleolus) was selected for each study participant. The players’ positions for the muscle strength assessment are presented in Figure 1.

For each movement, a set of three repetitions for each movement was selected for testing. The order established for testing the maximal muscle strength of right knee extension (RKE), left knee extension (LKE), right knee flexion (RKF) and left knee flexion (LKF) was allegorized to avoid learning bias. The BSB of the stronger leg relative to the weaker leg was calculated (Contreras-Díaz et al., 2023).

$$\text{Bilateral Strength Balance }\left(\%\right)=\frac{Strong\;Leg-Weak\;Leg}{Strong\;Leg\;}\;x\;100$$

### 2.5 Statistical analysis

Descriptive statistical models were used to calculate means and standard deviations (SD). Normal distribution of data was analyzed using the Shapiro-Wilk test. The BSB calculation is presented in percentages (%). Independent samples t-test and standardized mean differences (independent samples effect size) were used to analyze differences in knee flexor and extensor muscle strength between the dominant and non-dominant limbs during concentric and eccentric contraction modes. The criteria for interpreting the magnitude of the modified Cohen-Hopkins effect size (ES) were as follows: null (<0.20), small (0.2–0.59), moderate (0.60–1.19), large (1.20–2.00), very large (2.00–4.00), and extremely large (>4.0) (Hopkins, 2010). A 95% confidence interval was used in the statistical model calculations for its analysis. For this study, the statistical model was accepted for significance at *p* < 0.05. Statistical analyses were performed using JASP software (version 0.16.4).

## 3 Results

No significant differences (*p* > 0.05) were found in the comparison of independent means for maximum muscle strength with FEMD between the EXT and FLE knee groups in the CON and ECC phases. In addition, small ES (ES < 0.21) were reported for EXT in the ECC phase and the others were null (ES < 0.12), compared to the dominant and non-dominant lower limbs. Maximum muscle strength was greater in the knee extensor muscle group in the ECC and CON phases in the dominant leg. The knee flexor muscle group presented maximum strength in the CON phase in the non-dominant leg, and greater maximum strength was observed in the dominant leg in the ECC phase. In the bilateral strength balance comparison of indices, maximum strength measurements were obtained in the knee extensor muscle group and in the knee flexor muscles between 13.27% and 15.21%, in the concentric and eccentric phases, las presented in Table 2.

**TABLE 2:** Bilateral comparison of peak muscle strength in knee extension and flexion in concentric and eccentric phases in the dominant and non-dominant lower limb.

Muscle action	Phase	Mean ± SD (N)						*p*-Value	ES	BSB (%)
		Dominant			Non-Dominant					95%IC
Extension	Concentric	171.27	±	25.87	169.03	±	22.27	0.754	0.05	10.68 (15.35–6.019)
	Eccentric	313.88	±	71.18	294.15	±	68.60	0.354	0.21	13.80 (18.36–10.54)
Flexion	Concentric	77.76	±	15.47	78.63	±	10.52	0.825	0.04	15.21 (23.37–7.053)
	Eccentric	152.50	±	39.36	146.99	±	36.05	0.624	0.12	13.27 (18.75–7.806)

Newton; p-Value, significance level; SD, standard deviation; ES, Cohen’s d effect size; BSB, bilateral strength balance; 95%IC, Confidence interval.

Significant differences (*p* < 0.01) and small ES (ES < 3.32) were found in muscle maximum strength with FEMD measurements of the dominant and non-dominant lower extremity in the unilateral comparison of the extensor muscle and the flexor muscle group in the concentric and eccentric phase as presented in Table 3.

**TABLE 3:** Comparison of peak muscle strength in the dominant and non-dominant lower extremity in knee extension and flexion in concentric and eccentric phases.

Muscle action	Phase	Mean ± SD (N)						*p*-Value	ES
		Extension			Flexion				
Dominant	Concentric	171.27	±	25.87	77.76	±	15.47	0.001	2.75
	Eccentric	313.88	±	71.18	152.50	±	39.36	0.001	2.14
Non-Dominant	Concentric	169.03	±	22.27	78.63	±	10.52	0.001	3.32
	Eccentric	294.15	±	68.60	146.99	±	36.05	0.001	2.06

N: Newton; p-Value, significance level; SD, standard deviation; ES, Cohen’s d effect size.

## 4 Discussion

The purpose of our study was to primary objective of our study was to analyze the bilateral strength balance of the knee extensor and flexor muscles in female soccer players, secondly, to present an analysis of bilateral and unilateral maximum muscle strength of the knee extensor and flexor muscles. In this study, the bilateral strength balance comparison of indices, maximum strength measurements were obtained in the knee extensor muscle group and in the knee flexor muscles between 13.27% and 15.21%, in the concentric and eccentric phases. In addition, they reported that the peak muscular strength was greater for the knee extensor muscle group in the CON and ECC phases in the dominant leg, for the knee flexor muscle group there was greater maximum strength in the CON phase in the non-dominant leg and for the ECC phase the maximum strength was greater in the dominant leg. For measurements of maximum muscle strength of the dominant and non-dominant lower limb in the unilateral comparison of the extensor muscle group and the flexor muscle group in the concentric and eccentric phase, significant differences (*p* < 0.01) and a low ES (ES < 3.32) were found.

In a study Velázquez et al. (2020), they show that the difference in muscle strength between the dominant and non-dominant legs in non-injured soccer players is less than 12% in the knee flexor muscles, lower than our findings. Some studies suggest that these muscle strength imbalances are typical of sports training, considering that each sporting action or gesture mostly uses the dominant leg (Bishop et al., 2021). However, there is reported evidence that suggests that muscle strength asymmetry is not the direct cause of muscle injuries (Dos’Santos et al., 2018). Furthermore, the greater asymmetries reported in top division soccer players in contrast to lower division soccer players suggests that a high level of competition could favor lower limb muscle strength asymmetry (Ferreira et al., 2018). The results reported in this study do not agree with the background reported in the study by Fousekis et al. (2010), who indicated that prolonged activity in collaborative and opposition sports such as soccer can produce a lower level of bilateral lower limb asymmetry in the sport’s practitioners. In this study, higher values are reported in the bilaterality index of the knee flexor muscle group compared to the knee extensor muscle group, results that coincide with those reported in the study by Maly et al. (2016), which observed these results in professional soccer players from Brazil.

In the study Paravlic et al. (2024), similar results to those of this study were observed, reporting significantly higher values of strength in the extensor and flexor muscles of the knee in the dominant leg compared to the non-dominant in elite soccer players, which can be interpreted as adaptations of the game due to the physiological and anthropometric modifications produced as the percentage of muscle mass. Furthermore, this study found significant differences (*p* < 0.01) and very large effect sizes (ES ≤ 3.32) in measurements of peak muscle strength of the dominant and non-dominant lower limb when comparing the extensor muscle group and the flexor muscle group in the concentric and eccentric phase. Similar results were obtained in the study by Parpa and Michaelides (2022) in which significant differences in peak muscle strength of the knee extensor and flexor muscles were reported, which may be attributed to the high demand of sprinting and changing directions required in soccer. In another study by (Kumar & Kumar, 2020), similarities to our results were also found, reporting significant differences in greater muscle activation in the knee extensor muscles compared to the flexor muscles during kicking in soccer.

Our study reported similarities with the study (Holcomb et al., 2007), which reported a higher quadriceps hamstring ratio in the non-dominant lower group of female college soccer players. This could be explained because the non-dominant lower limb producing higher quadriceps hamstring ratio indices is directly associated with the fact that this leg is generally used more as a stabilizing leg, remaining stationary while the dominant would be the one hitting the ball (Brígido-Fernández et al., 2022).

This article is not without limitations, the research group was composed of second division footballers and there may be differences in the physical capacities of footballers from another division, categorization by position on the field has not been considered because the number of players per position was considered low, In addition, with a cross-sectional design and a small convenience sample size, longitudinal studies that evaluate the prediction of injury risk through the evaluation of bilateral strength balance should be presented.

Future research should consider the limitations of this study. We suggest initiating studies that analyze different athlete populations and competition levels to determine their maximal muscle strength and analyze bilaterality, as well as presenting longitudinal studies to evaluate injury risk prediction through bilateral strength balance assessment.

## 5 Conclusion

The results of our study that performed an analysis of bilateral strength balance in which lower indices are presented in the knee extensor group compared to the flexor group, these bilateral indices would reveal intrinsic and extrinsic risk factors for musculoskeletal injuries in the hamstring muscles and anterior cruciate ligament in professional soccer players. Furthermore, these results could be attributed to the fact that hamstring injury is the most common in football and most injuries in the hamstring muscle group occur more frequently and bilateral strength balance could be influenced by this situation. The evaluation of maximum muscle strength in knee extension and flexion in concentric and eccentric phases controlled FEMD, report that there is greater peak muscle force in the dominant leg for the extensor group and greater peak muscle force in the non-dominant group in the leg flexor group, in addition, there is a significant difference in the peak muscle strength of the extensor group compared to the flexor group. Periodic assessment of maximum muscle strength and bilateral strength analysis could reduce the possibility of lower limb muscle injury in female soccer players.

## Data Availability

The raw data supporting the conclusions of this article will be made available by the authors, without undue reservation.
